# Self‐evaluation of duration of adjuvant chemotherapy side effects in breast cancer patients: A prospective study

**DOI:** 10.1002/cam4.1687

**Published:** 2018-07-20

**Authors:** Danilo Galizia, Andrea Milani, Elena Geuna, Rossella Martinello, Celeste Cagnazzo, Manuela Foresto, Virginia Longo, Paola Berchialla, Gianfranca Solinas, Adele Calori, Bruna Grasso, Chiara Volpone, Gisella Bertola, Gisella Parola, Giancarla Tealdi, Piero Luigi Giuliano, Anna Maria Ballari, Massimo Aglietta, Filippo Montemurro

**Affiliations:** ^1^ Investigational and Clinical Oncology (INCO) Candiolo Cancer Institute‐FPO, IRCCS Candiolo Italy; ^2^ Clinical Research Office Candiolo Cancer Institute‐FPO, IRCCS Candiolo Italy; ^3^ Multidisciplinary Day Hospital Candiolo Cancer Institute‐FPO, IRCS Candiolo Italy; ^4^ AOU Città della Salute e della Scienza di Torino ‐ Presidio Sant’ Anna Turin Italy; ^5^ University of Turin Turin Italy; ^6^ AOU Maggiore della Carità ‐ Novara Novara Italy; ^7^ Ospedale Cardinal Massaia ‐ Asti Asti Italy; ^8^ Azienda Sanitaria Locale ASL CN2‐Alba‐Bra Alba Italy; ^9^ Azienda Sanitaria Locale Verbano Cusio Ossola ‐ Verbania Verbania Italy; ^10^ Ospedale Civile Ivrea Ivrea Italy; ^11^ Azienda ASO S. Croce e Carle ‐ Cuneo Cuneo Italy; ^12^ AOU Città della Salute e della Scienza di Torino ‐ Presidio Molinette COES Turin Italy; ^13^ Azienda AOU San Luigi Gonzaga ‐ Orbassano Turin Italy; ^14^ Medical Oncology Candiolo Cancer Institute‐FPO, IRCCS Candiolo Italy; ^15^ Department of Medical Oncology University of Turin Turin Italy

**Keywords:** adjuvant chemotherapy, breast cancer, chemotherapy‐related side effects, common toxicity criteria for adverse events, duration, patient‐reported outcomes

## Abstract

**Background:**

We recently reported that self‐evaluation of the incidence and severity of treatment‐related side effects (TSEs) using a National Cancer Institute (NCI) Common Terminology Criteria for Adverse Events (CTCAE) v4.0‐based questionnaire was feasible and more informative than doctor reports in patients undergoing standard adjuvant chemotherapy for operable breast cancer. Here, we compare self‐ and doctor‐evaluated day of onset and duration of TSEs in the same population.

**Patients and methods:**

Six hundred and four patients were enrolled at 11 sites in Italy. CTCAE v4.0 definitions of grade of severity of nausea, vomiting, constipation, anorexia, dysgeusia, diarrhea, fatigue, pain, paresthesia, and dyspnea were translated into Italian and rephrased. Questionnaires were administered after the first and third chemotherapy cycles. At each time‐point, information on TSEs was extracted from the medical charts and compared to patient questionnaires.

**Results:**

A total of 594 and 573 paired patient and doctor questionnaires were collected after cycles one and three, respectively. TSE duration was significantly longer when reported by patients compared to doctors for six and seven of ten items after cycles one and three, respectively. Due to the combined effect of doctor underreporting of TSE incidence and duration, the mean percentages of cycle days with TSEs were significantly higher for all ten items when based on patient reports. Day of onset could not be evaluated because of insufficient numbers of complete records.

**Conclusions:**

Self‐reporting TSE duration is feasible using a CTCAE‐derived questionnaire. As doctors tend to underestimate TSE incidence and duration, patient‐reported outcomes should be incorporated into clinical practice, perhaps using eHealth technologies, to harness their potential to better estimate total TSE burden.

## 1 INTRODUCTION

The collection and analysis of treatment‐related side effects (TSEs) are critical to the management of patients with cancer both in clinical trials and in daily practice.^1^, ^2^ Systems like the National Cancer Institute (NCI) Common Terminology Criteria for Adverse Events (CTCAE) are integral to clinical trials where measures of treatment‐related toxicity are critical for establishing the risk‐benefit ratio of new treatments. Interestingly, a number of papers have reported moderate to substantial underestimation of TSE reporting when investigator‐collected data are compared to corresponding data provided by patients using adapted questionnaires.^3^, ^4^, ^5^, ^6^, ^7^, ^8^ For this reason, the NCI has promoted the development of a “patient” version of the CTCAE (patient‐reported outcome (PRO)‐CTCAE), which is currently being translated and validated in different languages and integrated, together with more established PROs, in clinical trials.^9^, ^10^, ^11^, ^12^, ^13^

If patient‐ and doctor‐reported TSEs are discrepant in the prospective clinical trial context, it is likely that TSE underestimates are even greater in patients receiving standard treatments in clinical practice, as there is no standardized system to collect them. TSE underreporting may have consequences for quality of life and disease‐related outcomes.^8^, ^14^, ^15^

Therefore, we developed a CTCAE v4.0‐based ten‐item patient questionnaire by translating grade of toxicity definitions into Italian and administering it to a large cohort of patients with breast cancer receiving adjuvant chemotherapy at different Italian institutions.^16^ We showed that doctors underestimate TSE incidence and severity in clinical practice, with a more pronounced effect in high‐volume centers. However, our questionnaire also reported the day of onset and duration for each TSE considered, allowing us to here to analyze and compare these two patient‐ and doctor‐reported TSE dimensions.

## 2 PATIENTS AND METHODS

The details of our prospective trial evaluating a CTCAE v4.0‐based patient questionnaire are reported elsewhere.^16^ Our ten‐item paper questionnaire included nausea, vomiting, constipation, anorexia, dysgeusia, diarrhea, fatigue, pain (generic), paresthesia, and dyspnea. For each item, definitions and severity grades were translated into Italian from the CTCAE version 4.0. In addition to severity grade, the questionnaire contained fields to record the day of onset for each item (where the day of chemotherapy administration was day one), the duration in days, and persistence at the time of questionnaire administration (Figure S1). When the Italian translation of CTCAE version 4.0 became available and was endorsed by the Italian Association of Medical Oncology in mid‐2011, the questionnaire was rechecked and there were no translational discrepancies.^17^

Patients were instructed to complete the questionnaire at the end of the first cycle (usually on day one of the second cycle of planned chemotherapy) and at the end of the third cycle (usually on day one of the fourth cycle of planned chemotherapy). Dedicated nurses provided instructions on how to complete the questionnaires at the time of obtaining informed consent, with no further assistance given during the study. Patients were also provided with a diary to help record onset and duration of specific adverse events, but this was not part of the formal study materials. At each participating Institution, dedicated nurses extracted side effect information from the medical records of patients and filled in “doctor” questionnaires at matching time‐points. These questionnaires were collected exclusively by nurses and were not available to the treating doctors.

### 2.1 Statistical analysis

Medians and means are reported together with their ranges and standard errors, respectively. Paired medians and means were compared by the Wilcoxon signed‐rank test and by Student's *t* test for paired data. Proportions in unrelated samples were compared by the chi‐squared test. Statistical significance was set at *P *<* *0.05 (two‐tailed).

## 3 RESULTS

Of 604 patients with early breast cancer enrolled in the study at 11 Italian Institutions, three withdrew informed consent before the first cycle of chemotherapy. Patient demographics are summarized in Table 1. Overall, 596 and 581 patient questionnaires were collected after cycles one and three of adjuvant chemotherapy, respectively. Of these, 594 and 573 had a corresponding (doctor) questionnaire extracted from the medical charts at the same time‐points, respectively. In patients reporting TSEs, the average proportions of questionnaires with complete information on day of onset and duration were 70% and 95%, respectively (first questionnaire), and 75% and 95%, respectively (second questionnaire, Tables S1 and S2). Corresponding figures for doctor reports were 29% and 95%, respectively (first questionnaire), and 31% and 97%, respectively (second questionnaire, Tables S1 and S2).

**Table 1:** Patients demographics

Variable	Number	% or ranges
Median age in years	53.4	45.0‐62.7
Histology, N (%)		
Ductal	490	82
Lobular	54	9
Other	53	8
Missing	4	<1
Type of breast surgery		
Mastectomy	242	40
Breast‐conserving surgery	354	49
Missing	5	1
Type of axillary surgery		
Sentinel lymph node biopsy	268	45
Axillary dissection	328	55
Missing	5	1
Type of adjuvant chemotherapy		
FEC_90_ or FEC_100_	387	64
AC o EC	133	22
TC	81	14

FEC_90_, 5‐fluorouracil (600 mg/m^2^), epi‐doxorubicin (90 mg/m^2^), cyclophosphamide (600 mg/m^2^); FEC_100_, 5‐fluorouracil (500 mg/m^2^), epi‐doxorubicin (100 mg/m^2^), cyclophosphamide (500 mg/m^2^); AC, doxorubicin (60 mg/m^2^), cyclophosphamide (600 mg/m^2^); EC, epi‐doxorubicin (90 mg/m^2^), cyclophosphamide (600 mg/m^2^); TC, docetaxel (75 mg/m^2^), cyclophosphamide (600 mg/m^2^).

The median day of onset and median durations as reported by patients and described by doctors in the first and second questionnaires are summarized in Tables S3 and S4. As data on day of onset were missing in a high proportion of doctor questionnaires, patient and doctor reports of day of onset could not be formally compared.

The median duration values of each of the ten adverse events as reported by patients and as described by doctors after cycles one and three were only compared for pairs of charts where both patients and doctors reported each item as present (any grade). After cycle one, with the exception of vomiting, pain, neuropathy, and dyspnea, the median duration of symptoms was longer when reported by patients than doctors, with notable differences for nausea (4 vs 2 days; *P *<* *0.01), constipation (3 vs 1 days; *P *<* *0.01), anorexia (3 vs 1 days; *P *<* *0.01), diarrhea (3.5 vs 1 day; *P *=* *0.02), and fatigue (4 vs 1 days; *P *<* *0.01) (Table 2). Results were similar after the third cycle, with the difference that vomiting also lasted longer when reported by patients than doctors and that the difference in the duration of diarrhea was not statistically significant (Table 3).

**Table 2:** Summary of pairwise comparisons of median duration of TSEs after cycle one (first questionnaire)

Item	N paired questionnaires reporting the item^a^	Duration patient median (range)	Duration doctor median (range)	*P*
Nausea	175	4 (1‐21)	2 (1‐21)	<0.01
Vomiting	54	2 (1‐6)	1 (1‐10)	N.S.
Constipation	52	3 (1‐20)	1 (1‐14)	<0.01
Anorexia	34	3 (1‐15)	1 (1‐10)	<0.01
Dysgeusia	38	2 (1‐21)	1 (1‐20)	<0.01
Diarrhea	17	3.5 (1‐13)	1 (1‐7)	0.02
Fatigue	112	4 (1‐21)	1 (1‐14)	<0.01
Pain	28	1 (1‐15)	1 (1‐15)	NS
Paresthesia	13	1 (1‐6)	1 (1‐6)	NS
Dyspnea	11	1 (1‐6)	1 (1‐6)	NS

Number in parentheses indicate paired patient and doctor questionnaires available for the analysis of incidence.

NS, nonsignificant.

Numbers indicate the pairs of questionnaires where both the patient and the doctor indicated the occurrence of the side effect and for which information on duration was present.

**Table 3:** Summary of pairwise comparisons of median duration after cycle three (second questionnaire)

Item	N paired questionnaires reporting the item (grade ≥1)^a^	Duration patient median (range)	Duration doctor median (range)	*P*
Nausea	177	4 (1‐18)	2 (1‐21)	<0.01
Vomiting	53	2 (1‐8)	1 (1‐5)	<0.01
Constipation	54	3 (1‐20)	1 (1‐18)	<0.01
Anorexia	35	4 (1‐15)	1 (1‐15)	0.01
Dysgeusia	48	2 (1‐21)	1 (1‐21)	0.03
Diarrhea	12	3 (1‐12)	1 (1‐7)	0.06
Fatigue	103	4 (1‐40)	1 (1‐21)	<0.01
Pain	27	2 (1‐21)	1 (1‐21)	NS
Paresthesia	16	1 (1‐10)	1 (1‐10)	NS
Dyspnea	22	1 (1‐19)	1 (1‐18)	NS

a Numbers indicate the pairs of questionnaires where both the patient and the doctor indicated the occurrence of the side effect and for which information on duration was present.

**Figure 1:**
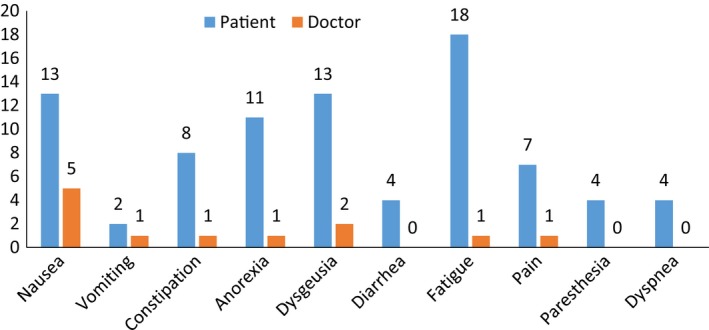
Percentage of cycle days with each adverse event as reported by patients and doctors. Decimals are rounded to the nearest unit. Source data are summarized in Table S5. All differences are statistically significant by Student's *t* test for paired data

To analyze the combined effect of differences in reporting incidence and duration, the mean percentages of cycle days with a certain side effect were calculated for each TSE (total days with the side‐effect/total days from the date of chemotherapy administration and that of data collection*100) as reported by patients and doctors. Here, “0” days described a TSE that either did not occur or that was missed by the patient or the doctor. Results for the first questionnaire are displayed in Figure 1 and in Table S5; the mean percentage of cycle days with each TSE was significantly higher when reported by patients compared to doctors. Similar results were obtained with the second questionnaire (data not shown).

## 4 DISCUSSION

Here, we exploited our CTCAE V4.0‐based questionnaire study to integrate data on time of onset and duration of ten common TSEs experienced by breast cancer patients treated with adjuvant chemotherapy in clinical practice.

In our prior publication comparing patient‐ and doctor‐reported TSE incidence and severity, we described a high rate of questionnaire completion, except for the field indicating persistence of the TSEs at the time of the visit.^16^ When reviewing the dataset, despite a high completion rate, a substantial number of “day of onset” entries in both the patient and doctor questionnaires were “0” values. As the day of onset had to be counted from the day of the last chemotherapy administration (day one), we considered all the 0 values as incorrect and treated them as missing values. Consequently, for this toxicity dimension, we were unable to perform a formal statistical comparison of paired patient and doctor questionnaires for all the items because due to insufficient paired data. The descriptive analysis reported in Tables S3 and S4 suggested no major differences in the median day of onset but a larger variability in patient‐reported nausea, vomiting, constipation, anorexia, and dysgeusia.

Conversely, data on TSE duration were of better quality and reported in over 90% of patient and doctor questionnaires. In paired comparisons, patients reported longer duration after the first and third cycles for six and seven of the ten items, respectively. By combining information on incidence and duration, we determined that the mean percentage of cycle days with a certain toxicity was significantly higher in patient than doctor reports. These results indicate major discrepancies in the subjective experience of patients given the opportunity to self‐collect TSE to medical chart reports during routine visits at oncology centers compared to doctor reports.

Nevertheless, our data suggest that longitudinal TSE evaluations by patient self‐administered questionnaires are feasible and could improve the management of patients undergoing adjuvant chemotherapy for breast cancer.^18^, ^19^

The metrics and tools available to longitudinally evaluate side effects to dissect and exploit the different TSE dimensions in clinical trials evaluating newer treatments have recently been reviewed and discussed.^20^, ^21^ This issue is becoming particularly relevant to new treatment paradigms such as the chronic administration of low‐dose chemotherapy or use of biologicals. Longitudinal assessment requires several TSE dimensions to be collected during treatment to feed databases that can generate appropriate metrics. In this respect, a tool like our questionnaire or the more standardized PRO‐CTCAE with integrated day of onset and duration could provide such data. Although emphasis is often given to acute toxicities from cytotoxic chemotherapy, longitudinal assessment of the dimensions of timing of onset and duration may improve TSE management in routine clinical practice. Questionnaires like or own could also be converted into Web‐based tools or smartphone “eHealth” applications for the patient to complete at home, with remote access by medical staff. Some nonrandomized studies of electronic TSE data capture have been conducted, highlighting the promising potential of this approach.^22^, ^23^, ^24^ Importantly, a recently published randomized trial showed that advanced cancer patients undergoing treatment managed with a system integrating a Web‐based version of the PRO‐CTCAE and remote control with appropriate actions when needed (ie, patients reporting significant worsening of a certain TSE) had, compared with usual care, improved overall survival.^15^

In conclusion, here we confirm that patients with breast cancer undergoing adjuvant chemotherapy can self‐assess and report the day of TSE onset and describe their duration. The duration of most TSEs was longer when reported by patients rather than doctors, and the combined effect of longer duration and underreporting was an increase in the percentage of cycle days with all TSEs. We are now implementing an electronic version of our questionnaire in the routine management of patients with breast cancer undergoing adjuvant chemotherapy at our institution.

## CONFLICT OF INTEREST

Speaker's honoraria: Astra Zeneca, Novartis, Roche; Travel grants: Astra Zeneca, Roche; Consulting or Advisory Role: Bristol‐Myers Squibb, Merck, Roche; Travel, Accommodations, Expenses: Bristol‐Myers Squibb; All remaining authors have declared no conflict of interest.
