# Supplementary material for: Self‐evaluation of duration of adjuvant chemotherapy side effects in breast cancer patients: A prospective study
Source: Cancer Med. 2018 Jul 20;7(9):4339–44. doi: 10.1002/cam4.1687 (PMC6144000; doi:10.1002/cam4.1687)
Supplement: Supplementary file 1 [file CAM4-7-4339-s001.pdf]

1 Nausea

1) Have you felt nauseated?	No ☐		If no, go to item 2
	Yes ☐		If yes, chose one of the definitions below*
G1	☐	I felt nauseated, but I was able to eat and drink as usual	
G2	☐	I felt nauseated and, because of that, I ate and drank less than usual	
	☐	Because of nausea, I was given intravenous fluids, but for less than one day	
G3	☐	Because of nausea I was not able to eat and drink for several days: for this reason, I was given prolonged (more than one day) intravenous fluids and/or artificial nutrition	
Day nausea started (consider as day 1 the day when chemotherapy was administered)	_______	Duration in days______ or persistent; Yes No	

*Please, choose which of the definitions below best represents the worst experience related to nausea occurring at any time during the interval between the previous cycle of chemotherapy and today.

2) Vomiting

1) Have you vomited?	No ☐		If no, go to item 3
	Yes ☐		If yes, chose one of the definitions below*
G1	☐	I vomited 1 or 2 times in one day	
G2	☐	I vomited 3 to 5 times in one day	
G3	☐	I vomited 6 or more times in one day	
	☐	Because of vomiting, I was given intravenous fluids and/or artificial nutrition	
G4	☐	Because of vomiting, I needed urgent hospitalization and I felt that my life was in danger	
Day of the first episode of vomiting (consider as day 1 the day when chemotherapy was administered)	_______	Duration in days______ or persistent; Yes No	

*Please, choose which of the definitions below best represents the worst experience related to vomiting occurring at any time during the interval between the previous cycle of chemotherapy and today.
