# Supplementary material for: Self‐evaluation of duration of adjuvant chemotherapy side effects in breast cancer patients: A prospective study
Source: Cancer Med. 2018 Jul 20;7(9):4339–44. doi: 10.1002/cam4.1687 (PMC6144000; doi:10.1002/cam4.1687)
Supplement: Supplementary file 2 [file CAM4-7-4339-s002.docx]

Table S1. Completeness of fields regarding duration and day of onset (First questionnaire)

| Items | Patients |  |  |  |  | Doctors |  |  |  |
| --- | --- | --- | --- | --- | --- | --- | --- | --- | --- |
|  | N of  quest.* | N with TSE (%) | Day Onset  N (%) | Duration  N (%) |  | N of  quest. | N with TSE  (%) | Day Onset | Duration |
| Nausea | 587 | 405 (69) | 311 (77) | 394 (97) |  | 545 | 220 (40) | 65 (46) | 206 (94) |
| Vomiting | 594 | 145 (24) | 113 (78) | 138 (95) |  | 573 | 62 (11) | 21 (34) | 59 (95) |
| Constipation | 586 | 304 (52) | 216 (71) | 291 (96) |  | 555 | 66 (12) | 12 (18) | 61 (92) |
| Anorexia | 593 | 319 (54) | 240 (75) | 308 (97) |  | 566 | 42 (7) | 14 (33) | 40 (95) |
| Dysgeusia | 578 | 295 (51) | 205 (70) | 277 (94) |  | 570 | 49 (9) | 12 (24) | 46 (94) |
| Diarrhea | 582 | 93 (16) | 72 (77) | 88 (95) |  | 581 | 28 (5) | 14 (50) | 25 (89) |
| Fatigue | 576 | 443 (77) | 310 (70) | 416 (94) |  | 549 | 136 (25) | 24 (18) | 126 (93) |
| Pain | 583 | 212 (36) | 153 (71) | 201 (95) |  | 595 | 53 (89) | 15 (28) | 50 (94) |
| Neuropathy | 586 | 134 (23) | 76 (57) | 123 (92) |  | 591 | 17 (3) | 5 (29) | 17 (100) |
| Dyspnea | 583 | 148 (25) | 98 (66) | 138 (93) |  | 587 | 13 (2) | 4 (13) | 13 (100) |
| Mean completion rates |  |  | 70% | 95% |  |  |  | 29% | 95% |

TSE, treatment-related side effect

*Number of questionnaires reporting data on the occurrence of each SE.
