# Supplementary material for: Self‐evaluation of duration of adjuvant chemotherapy side effects in breast cancer patients: A prospective study
Source: Cancer Med. 2018 Jul 20;7(9):4339–44. doi: 10.1002/cam4.1687 (PMC6144000; doi:10.1002/cam4.1687)
Supplement: Supplementary file 3 [file CAM4-7-4339-s003.docx]

Table S2. Completeness of fields regarding duration and day of onset (Second questionnaire)

| Items | Patients |  |  |  |  | Doctors |  |  |  |
| --- | --- | --- | --- | --- | --- | --- | --- | --- | --- |
|  | N of  quest.* | N with TSE (%) | Day Onset  N (%) | Duration  N (%) |  | N of  quest. | N with TSE  (%) | Day Onset | Duration |
| Nausea | 561 | 407 (72) | 316 (78) | 395 (97) |  | 530 | 199 (38) | 75 (38) | 198 (99) |
| Vomiting | 568 | 118 (21) | 93 (79) | 118 (100) |  | 556 | 59 (11) | 31 (52) | 59 (100) |
| Constipation | 563 | 286 (51) | 206 (72) | 224 (78) |  | 545 | 63 (12) | 18 (29) | 63 (100) |
| Anorexia | 571 | 300 (52) | 221 (74) | 285 (95) |  | 546 | 39 (7) | 14 (36) | 39 (100) |
| Dysgeusia | 552 | 324 (59) | 230 (71) | 308 (95) |  | 551 | 56 (10) | 14 (25) | 52 (93) |
| Diarrhea | 561 | 77 (14) | 68 (88) | 77 (100) |  | 561 | 20 (4) | 6 (30) | 20 (100) |
| Fatigue | 561 | 442 (79) | 316 (71) | 408 (92) |  | 532 | 124 (23) | 26 (21) | 119 (96) |
| Pain | 563 | 191 (34) | 143 (75) | 180 (94) |  | 518 | 46 (9) | 13 (28) | 45 (98) |
| Neuropathy | 567 | 120 (21) | 74 (62) | 111 (92) |  | 571 | 20 (4) | 5 (25) | 18 (90) |
| Dyspnea | 564 | 160 (28) | 104 (65) | 145 (91) |  | 562 | 27 (5) | 6 (22) | 25 (93) |
| Mean completion rates |  |  | 75% | 93% |  |  |  | 31% | 97% |

TSE, treatment-related side effect

*Number of questionnaires reporting data on the occurrence of each TSE
