# Supplementary material for: Self‐evaluation of duration of adjuvant chemotherapy side effects in breast cancer patients: A prospective study
Source: Cancer Med. 2018 Jul 20;7(9):4339–44. doi: 10.1002/cam4.1687 (PMC6144000; doi:10.1002/cam4.1687)
Supplement: Supplementary file 4 [file CAM4-7-4339-s004.docx]

Table S3. Summary of duration of treatment-related side effects as reported by patients and doctors in the first questionnaire

|  | Patients | | Doctors | |
| --- | --- | --- | --- | --- |
| Item | Median day of onset (range) | Median duration (range) | Median day of onset (range) | Duration mean (Range) |
| Nausea | 1 (1-14) | 3 (1-21) | 1 (1-9) | 2 (1-21) |
| Vomiting | 1 (1-13) | 2 (1-10) | 1 (1-4) | 1 (1-10) |
| Constipation | 2 (1-22) | 3 (1-21) | 2 (1-8) | 1 (1-14) |
| Anorexia | 2 (1-20) | 3 (1-21) | 1 (1-7) | 1 (1-10) |
| Dysgeusia | 2 (1-20) | 5 (1-21) | 2 (1-4) | 1 (1-20) |
| Diarrhea | 3 (1-20) | 4 (1-19) | 3.5 (1-16) | 1 (1-10) |
| Fatigue | 2 (1-20) | 4 (1-28) | 2 (1-15) | 1 (1-14) |
| Pain | 3 (1-19) | 3 (1-18) | 3 (1-17) | 1 (1-15) |
| Paresthesia | 3 (1-22) | 2 (1-28) | 4 (1-15) | 1 (1-6) |
| Dyspnea | 2 (1-18) | 2.5 (1-21) | 3 (1-15) | 1 (1-7) |
