# Supplementary material for: Self‐evaluation of duration of adjuvant chemotherapy side effects in breast cancer patients: A prospective study
Source: Cancer Med. 2018 Jul 20;7(9):4339–44. doi: 10.1002/cam4.1687 (PMC6144000; doi:10.1002/cam4.1687)
Supplement: Supplementary file 6 [file CAM4-7-4339-s006.docx]

Table S5. Percentage of cycle days with a TSE in patient and doctor question aires

| Item | Patient  Mean % (SE) | Doctor  Mean % (SE) | P* |
| --- | --- | --- | --- |
| Nausea | 13.4 (16.0) | 4.6 (9.0) | <0.01 |
| Vomiting | 2.3 (5.5) | 1.0 (3.8) | <0.01 |
| Constipation | 7.8 (13.0) | 1.4 (5.4) | <0.01 |
| Anorexia | 11.0 (16.8) | 0.6 (3.1) | <0.01 |
| Dysgeusia | 13.3 (22.0) | 0.9 (5.9) | <0.01 |
| Diarrhea | 4.2 (13.2) | 0.5 (3.0) | <0.01 |
| Fatigue | 18.0 (23.3) | 0.6 (3.1) | <0.01 |
| Pain | 7.0 (14.6) | 0.8 (3.8) | <0.01 |
| Paresthesia | 3.91 (12.3) | 0.3 (2.3) | <0.01 |
| Dyspnea | 5.2 (15.0) | 0.3 (2.5) | <0.01 |

Student’s T test for paired data
